# Supplementary material for: Aspartyl Protease Inhibitors as Anti-Filarial Drugs
Source: Pathogens. 2022 Jun 18;11(6):707. doi: 10.3390/pathogens11060707 (PMC9227574; doi:10.3390/pathogens11060707)
Supplement: Supplementary file 1 [file pathogens-11-00707-s001.zip › Supplementary Figure S1.pdf]

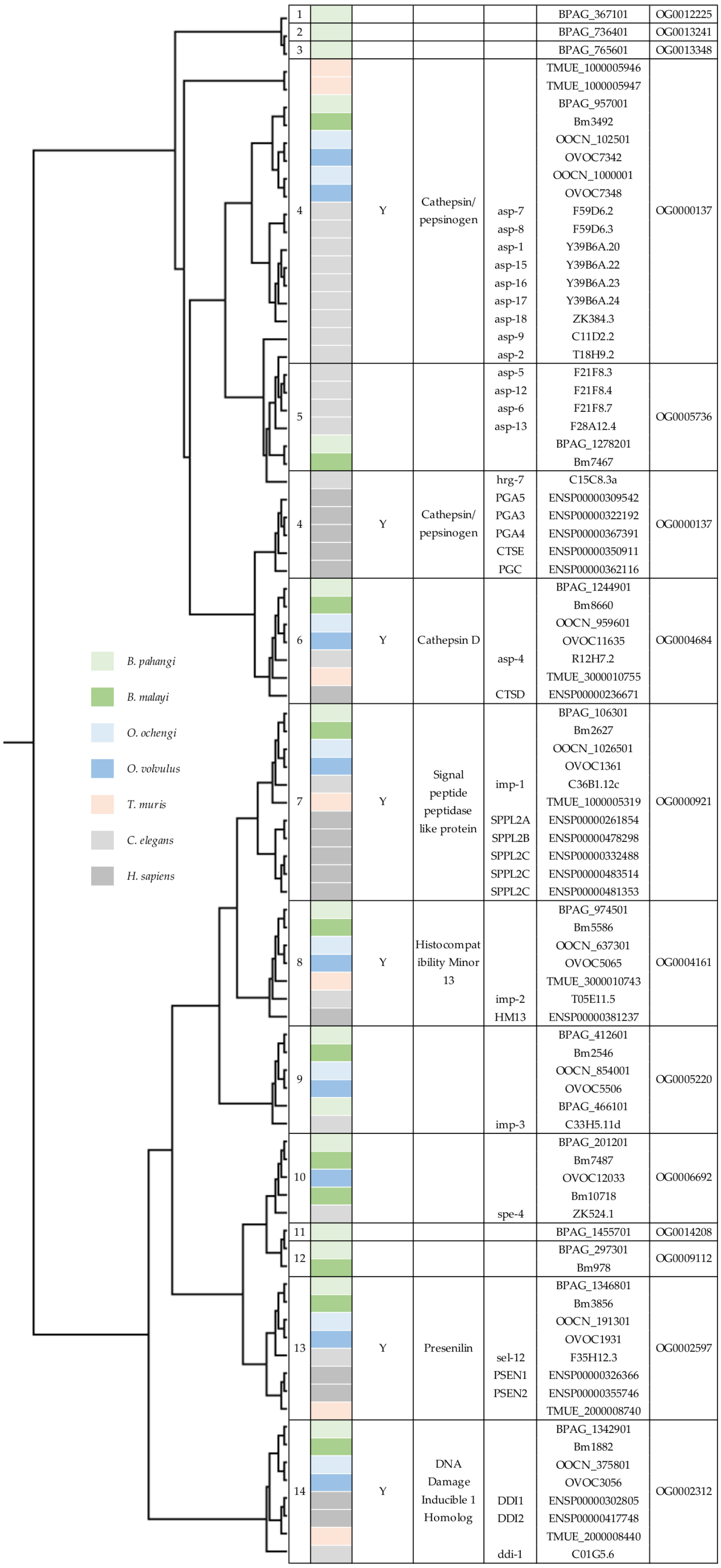

	Species	All conserved?	Annotation	Gene symbol	Protein ID	Orthogroup
1					BPAG_367101	OG0012225
2					BPAG_736401	OG0013241
3					BPAG_765601	OG0013348
4		Y	Cathepsin/ pepsinogen		TMUE_1000005946	OG0000137
					TMUE_1000005947	
					BPAG_957001	
					Bm3492	
					OOCN_102501	
					OVOC7342	
					OOCN_1000001	
					OVOC7348	
				asp-7	F59D6.2	
				asp-8	F59D6.3	
				asp-1	Y39B6A.20	
				asp-15	Y39B6A.22	
				asp-16	Y39B6A.23	
				asp-17	Y39B6A.24	
				asp-18	ZK384.3	
				asp-9	C11D2.2	
				asp-2	T18H9.2	
5				asp-5	F21F8.3	OG0005736
				asp-12	F21F8.4	
				asp-6	F21F8.7	
				asp-13	F28A12.4	
					BPAG_1278201	
					Bm7467	
4		Y	Cathepsin/ pepsinogen	hrg-7	C15C8.3a	OG0000137
				PGA5	ENSP00000309542	
				PGA3	ENSP00000322192	
				PGA4	ENSP00000367391	
				CTSE	ENSP00000350911	
				PGC	ENSP00000362116	
6		Y	Cathepsin D		BPAG_1244901	OG0004684
					Bm8660	
					OOCN_959601	
					OVOC11635	
				asp-4	R12H7.2	
					TMUE_3000010755	
				CTSD	ENSP00000236671	
7		Y	Signal peptide peptidase like protein		BPAG_106301	OG0000921
					Bm2627	
					OOCN_1026501	
					OVOC1361	
				imp-1	C36B1.12c	
					TMUE_1000005319	
				SPPL2A	ENSP00000261854	
				SPPL2B	ENSP00000478298	
				SPPL2C	ENSP00000332488	
				SPPL2C	ENSP00000483514	
				SPPL2C	ENSP00000481353	
8		Y	Histocompat ibility Minor 13		BPAG_974501	OG0004161
					Bm5586	
					OOCN_637301	
					OVOC5065	
					TMUE_3000010743	
				imp-2	T05E11.5	
				HM13	ENSP00000381237	
9					BPAG_412601	OG0005220
					Bm2546	
					OOCN_854001	
					OVOC5506	
					BPAG_466101	
				imp-3	C33H5.11d	
10					BPAG_201201	OG0006692
					Bm7487	
					OVOC12033	
					Bm10718	
				spe-4	ZK524.1	
11					BPAG_1455701	OG0014208
12					BPAG_297301	OG0009112
					Bm978	
13		Y	Presenilin		BPAG_1346801	OG0002597
					Bm3856	
					OOCN_191301	
					OVOC1931	
				sel-12	F35H12.3	
				PSEN1	ENSP00000326366	
				PSEN2	ENSP00000355746	
					TMUE_2000008740	
14		Y	DNA Damage Inducible 1 Homolog		BPAG_1342901	OG0002312
					Bm1882	
					OOCN_375801	
					OVOC3056	
				DDI1	ENSP00000302805	
				DDI2	ENSP00000417748	
					TMUE_2000008440	
				ddi-1	C01G5.6	

Supplementary Figure S1: Sequence based-clustering of proteins from 14 aspartic protease orthologous protein families (OPFs) identified in *B. pahangi*
